# Metabolomic Analysis of Two *Parmotrema* Lichens: *P. robustum* (Degel.) Hale and *P. andinum* (Mull. Arg.) Hale Using UHPLC-ESI-OT-MS-MS

**DOI:** 10.3390/molecules22111861

**Published:** 2017-10-30

**Authors:** Alfredo Torres-Benítez, María Rivera-Montalvo, Beatriz Sepúlveda, Olivio N. Castro, Edgar Nagles, Mario J. Simirgiotis, Olimpo García-Beltrán, Carlos Areche

**Affiliations:** 1 Facultad de Ciencias Naturales y Matemáticas, Universidad de Ibagué, Carrera 22 calle 67, Ibagué 730001, Colombia; alfredo.torres@unibague.edu.co (A.T.-B.); maria.rivera@unibague.edu.co (M.R.-M.); edgar.nagles@unibague.edu.co (E.N.); 2 Departamento de Ciencias Químicas, Universidad Andrés Bello, Campus Viña del Mar, Quillota 980, Viña del Mar 2520000, Chile; bsepulveda@uc.cl; 3 Departamento de Química, Pontificia Universidad Católica del Perú, Lima 15088, Peru; ocastro@pucp.pe; 4 Instituto de Farmacia, Facultad de Ciencias, Universidad Austral de Chile, Campus Isla Teja 5090000, Valdivia, Chile; mario.simirgiotis@uach.cl or mario.simirgiotis@gmail.com; 5 Center for Interdisciplinary Studies on the Nervous System, Universidad Austral de Chile, Campus Isla Teja 5090000, Valdivia, Chile; 6 Departamento de Química, Facultad de Ciencias, Universidad de Chile, Casilla 653, Santiago 7800024, Chile

**Keywords:** electrospray, lichens, metabolomic, *Parmotrema*, UHPLC-MS-MS, orbitrap

## Abstract

Lichens are symbiotic associations of fungi with microalgae and/or cyanobacteria. Lichens belonging to the Parmeliaceae family comprise 2700 species of lichens, including the *Parmotrema* genus which is composed of 300 species. The metabolites of this genus include depsides, depsidones, phenolics, polysaccharides, lipids, diphenylethers and dibenzofurans, which are responsible for the biological activities reported including antidiabetic, antihelmintic, anticancer, antioxidant, antibacterial, anti-inflammatory, antimitotic, antitumoral, antifungal, and antioxidant enzyme inhibitory. Due to scarce knowledge of metabolomic profiles of *Parmotrema* species (*P. andinum* and *P. robustum*), a full metabolome study based on ultra-high performance liquid chromatography- diode array detector-electrospray ionization-quadrupole-orbitrap-mass-spectrometry (UHPLC-DAD-ESI-Q-orbitrap MS) was performed for a comprehensive characterization of their substances. From the methanolic extracts of these species, a total of 54 metabolites were identified for the first time using this hyphenated technique, including thirty compounds in *P. andinum*, and thirty-seven in *P. robustum*. Moreover, two compounds were not identified as known compounds, and could be new structures, according to our data. This report shows that this technique is effective and accurate for rapid chemical identification of lichen substances and the compounds identified could serve as chemotaxonomic markers to differentiate these ruffle lichens.

## 1. Introduction

Lichens, by definition, correspond to a symbiotic association between a fungus and one or more photosynthetic autotrophic organisms that may be a green algae or a cyanobacterium, resulting in a morphologically different thallus to each of its components as a totally new morphological entity [1] Recently it was discovered that a third party, such as basidiomicete yeast, can also be a component of lichens [2]. In the Colombian Andean region, 1396 species of lichens have been found distributed in 295 genera and 88 families, representing 89% of the species, 95% of the genera and 93% of the families. The richest families are Parmeliaceae, Graphidaceae, Physciaceae, Thelotremataceae and Cladoniaceae and the most diversified genera are represented by *Cladonia*, *Parmotrema*, *Hypotrachyna*, *Usnea*, *Arthonia*, *Porina*, *Lecanora*, *Leptogium* and *Sticta* [3]. Some 215 genera of lichens have been reported in Peru; The genus with the highest number of recorded species is *Cladonia*, followed by *Hypotrachyna* and *Heterodermia*, and the species with the greatest presence are *Chrysothrix candelaris* and *Cladonia melanopoda* [3].

The genus *Parmotrema* (spp.) is characterized by having flat lobes of the thallus growing along the substrate, often largely adhered to it. The thallus shows similar structures to threads, more or less branched, in the ventral face (rhizinas) or in the margin (cilia). Rhizines are not dichotomically branched. The lobes of the thallus have a size of between 0.2 cm and more than 1 cm wide, with colorless, simple or bacillary spores and a ventral face devoid of veins [1]. *Parmotrema* lichens are difficult to differentiate since they are similar in appearance [4,5,6,7,8], thus, the chemotaxonomical differentiation of these lichens is very important. Some chemical studies of *Parmotrema* species have been reported previously and some compounds were identified [5,9,10,11,12,13,14,15,16,17,18]. The compound 2-methylene-3-(*R*)-hydroxynonadecanoic acid was reported from *P. xanthinum* [5,12,13], while praesorediosic acid and protopraesorediosic acid were reported from *P. praesorediosum* [16,17], and methyl β-orcinolcarboxylate, atranorin, isolecanoric acid and lecanoric acid from *P. tinctorum* [18]. Furthermore, the depside 4-O-demethylmicrophyllinic acid was reported from *P. demethylmicrophyllinicum* [9,10], and protocetraric acid was reported from *P. dilatatum* [5,12,13]. Then consalazinic acid was found in *P. subisidiosum*, malonprotocetraric acid in *P. conformatum* [14], the depside β-alectoronic acid was reported from *Parmotrema* sp., and finally salazinic acid, methyl orsellinate and orsellinic acid were reported to occur in *P. stuppeum* [15].

Regarding the biological activity of *Parmotrema* species, several organic extracts of *P. grayana*, *P. pseudotinctorum*, *P. praesorediosum*, *P. stuppeum*, *P. tinctorum*, and *Parmotrema* sp. have been assayed for antidiabetic, antihelmintic, anticancer, antioxidant, antibacterial, anti-inflammatory, antimitotic, antitumoral, antifungal, and enzyme inhibitory activity [11].

High performance liquid chromatography-tandem mass spectrometry (HPLC-MS-MS) is a powerful technique that combines liquid chromatography with mass analyses and whose application is the detection and identification of metabolites in complex extracts, including unknown metabolites, based on its fragmentation patterns. A pioneering work by Leuckert and Holzmann [19] detected lichen substances using fast atom bombardment MS-MS and identified usnic acid, diffractaic acid, gyrophoric acid, lecanoric acid, orsellinic acid, ovoic acid, thamnolic acid, hypothamnolic acid, divaricatic acid, fumarprotocetraric acid, protocetraric acid, homosekikaic acid and sekikaic acid on the following lichens: *Alectoria ochroleuca*, *Umbilicaria torrefacta*, *Thamnolia vermicularis*, *Ophioparma ventosa*, *Cladonia cryptochlorophaea* and *Cladonia rei* [19]. Later, Parrot et al. [20] reported a study of eight chemotypes of *Ramalina siliquosa* using LC-ESI-MS-MS and identified ten lichen substances: conhypoprotocetraric acid, salazinic acid, peristictic acid, cryptostictic acid, protocetraric acid, stictic acid, norstictic acid, hypoprotocetraric acid, 4-O-demethylbarbatic acid, usnic acid. In another report, β-orcinol, orsenillic acid, choline sulphate, roccellic acid, montagnetol, lecanoric acid, erythrin, lepraric acid and acetylportentol were identified based on the HPLC–MS–MS approach in nine lichens belonging to the *Lichina*, *Collema* and *Roccella* genera [21]. Afterwards, Le Pogam et al. [22] proposed the rapid identification of lichen extracts using laser desorption/ionization time of flight mass spectrometry instead of electrospray ionization. The analyzed samples with this MS technique were *Diploicia canescens*, *Evernia prunastri*, *Ophioparma ventosa*, *Pseudevernia furfuracea*, *Roccella fuciformis*, *Xanthoria parietina*, Cladonia portentosa, *flavocetraria nivalis*, *Lecidella asema*, *Ramalina siliquosa*, *Vulpicida pinastri* and *Usnea filipendula*. However, only 2 to 5 compounds were reported in each studied species in general [22]. Finally, the lichens *Parmotrema grayana* and *Heterodermia obscurata* were studied using high performance liquid chromatography- electrospray ionization-quadrupole-time of flight-mass-mass spectrometry (HPLC-ESI-Qq-TOF-MS-MS) on negative ion mode and fifteen compounds were detected and identified from the organic extracts [23].

The Q-Exactive Focus is a hybrid high-resolution mass spectrometer used to detect and quantify small organic compounds. The hyphenated Q-Exactive Focus instrument is an HRAM instrument (high resolution accurate mass) which combines UHPLC-DAD (UHPLC-diode array detection) with an orbital trap (orbitrap), a quadrupole (Q) and a high-resolution collision cell (HCD), which allows high resolution diagnostic MS fragments [22,23,24,25,26]. In a continuation of our research on the identification of lichen substances [25,26], we have selected two unstudied *Parmotrema* lichens for chemotaxonomic fingerprinting and describe the full comprehensive phytochemical profile of *P. andinum* and *P. robustum* for the first time, based on UHPLC-DAD coupled with high resolution electrospray ionization tandem mass spectrometry (UHPLC-Q-Orbitrap-HRMS). The profiles serve as fingerprints to differentiate these lichens since the ruffle lichens are difficult to differentiate.

## 2. Results and Discussion

Electrospray orbitrap emerged as a very fast and versatile tool for the rapid identification of lichens [25,26]. Thus, two *Parmotrema* species were selected, *P. andinum* from Ancash, Peru and *P. robustum* from Colombia, in order to determine their metabolomic profiles and chemical fingerprints in order to differentiate them since the ruffle lichens are similar in appearance and very difficult to differentiate [4,6,8,27]. Below is the detailed explanation of the rapid metabolome analysis of the aforementioned unstudied *Parmotrema* species using this HRAM technique.

### 2.1. Parmotrema andinum

**Figure 1:**
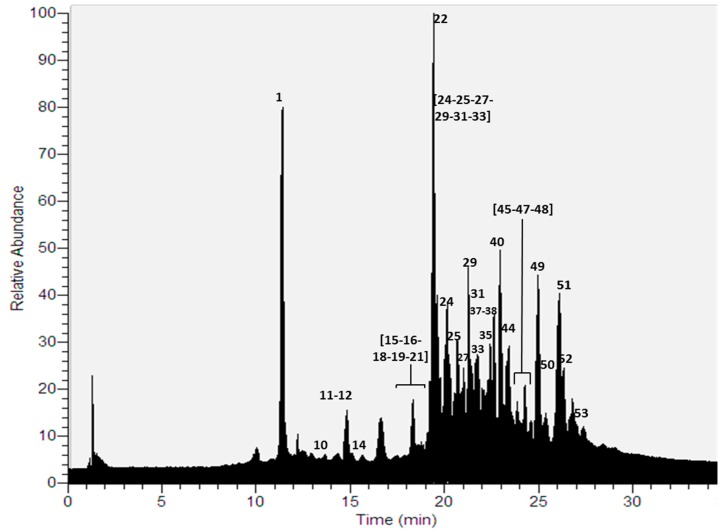
UHPLC Chromatogram of *P. andinum*.

Thirty compounds (Figure 1) were detected for the first time in a methanolic extract using UHPLC-ESI-MS-MS in negative mode (Table 1, Table S1). The identified compounds were mainly depsides, depsidones, lipids, aromatics, diphenylethers and dibenzofurans [9,10,22,23,24,25,26].

***Depsides***: Six depsides (peaks 18, 24, 33, 44, 45 and 51) were identified using a combination of diode array detection and high-resolution tandem mass spectrometry. Peak 18 was identified as lecanoric acid, which showed an [M − H]^−^ ion at *m*/*z* 317.0668. Major diagnostic daughter MS ions of lecanoric acid were [M − H − C_8_H_6_O_3_]^−^, [M − H − C_8_H_8_O_4_]^−^ and [C_7_H_7_O_2_]^−^ (167.0343, 149.0237 and 123.0444 a.m.u., respectively) [25]. Peak 24 was identified as decarboxythamnolic acid (molecular anion at *m*/*z* 375.0724), whose fragmentation produced diagnostic MS ions at *m*/*z* 209.0450, 167.0345 and 139.0394. Peak 33 was assigned to evernic acid, showing a molecular anion at *m*/*z* 331.0825. Its fragmentation produced ions at *m*/*z* 167.0345, 149.0238, and 123.0444 a.m.u. Peak 44, with an [M − H]^−^ ion at *m*/*z* 429.1922, was identified as 2-O-methylstenosporic acid. The parent ion produced major diagnostic MS ions at *m*/*z* 223.0972 [M − H − C_11_H_12_O_3_]^−^, and 179.1072 [C_9_H_11_O_2_]^−^ confirming this depside. Peak 45 presented a pseudomolecular ion at *m*/*z* 359.1138, which produced fragmented ions at *m*/*z* 181.0501, 163.0394 and 137.0600, and thus, was identified as barbatic acid. Peak 51 was identified as atranorin, which showed an [M − H]^−^ ion at *m*/*z* 373.0930. The major diagnostic daughter ions were at *m*/*z* 177.0190 and 163.0386 a.m.u.

***Depsidones***: Seven depsidones corresponding to peaks 11, 15, 16, 22, 25, 35, and 48 were identified using UHPLC-DAD and HRMS-MS analysis [25]. Peak 11 was identified as salazinic acid, which showed an [M − H]^−^ ion at *m*/*z* 387.0359. Its major diagnostic daughter ions were at *m*/*z* 243.0378, 227.0343, 151.0394 and 121.0291 a.m.u. Peaks 15 and 16 were identified as stictic acid and connorstictic acid, which showed [M − H]^−^ ions at *m*/*z* 385.0568 and 373.0568 respectively. The major diagnostic daughter ions were at *m*/*z* 341.0668, 297.0760, 267.0297 and 165.0544 a.m.u. for stictic acid, while for connorstictic acid ions they were at *m*/*z* 329.0665, and 181.0554 a.m.u. Peak 22, with an [M − H]^−^ pseudomolecular ion at *m*/*z* 371.0411, was identified as substictic acid, which showed diagnostic daughter ions at *m*/*z* 327.0512, and 255.0660. Hypoconstictic acid was identified as peak 25 (molecular anion at *m*/*z* 387.0724). The fragmentation of peak 25 produced ions at *m*/*z* 343.0825, and 299.0921. Peak 35, with an [M − H]^−^ ion at *m*/*z* 551.1194, was identified as furfuric acid. The parent ion produced major diagnostic MS ions at *m*/*z* 359.1130, 179.0345, 163.0394 and 137.0601 confirming this compound. Finally, peak 48 was identified as lobaric acid (molecular anion at *m*/*z* 455.1712) [26]. The fragmentation of peak 48 also produced ions at *m*/*z* 411.1815 [M − H − CO_2_]^−^, 367.1909 [M − H − 2CO_2_]^−^, 352.1681 [M − H − 2CO_2_ − CH_3_]^−^, and 296.1048 [M − H − 2CO_2_ − C_5_H_11_]^−^ confirming this depsidone.

***Lipids***: Six polyhydroxylated lipids were tentatively identified (peaks 21, 29, 38, 40, 49 and 53) using UHPLC–ESI–MS–MS analysis. Peak 21, with an [M − H]^−^ ion at *m*/*z* 403.3069, was tentatively identified as tetrahydroxydocosanoic acid. Peak 29 showed an [M − H]^−^ ion at *m*/*z* 517.3748 and was tentatively identified as pentahydroxyoxooctacosanoic acid. Peaks 38, 40 and 49 were tentatively identified as hydroxydioxohenicosanoic acid (C_21_H_38_O_5_), trioxohenicosanoic acid (C_21_H_36_O_5_) and dihydroxydioxononadecanoic (C_19_H_34_O_6_), which showed [M − H]^−^ ions at *m*/*z* 369.2649, 367.2492 and 357.2285 respectively. Finally, peak 53, with an [M − H]^−^ ion at *m*/*z* 295.1917, was tentatively identified as dihydroxyheptadecatrienoic acid (C_17_H_28_O_4_) [28].

***Diphenylethers***: Three diphenylethers (peak 27, 47 and 52) were detected in the methanolic extract using UHPLC-DAD-MS-MS analysis. Peak 27 was identified as loxodinol, which showed an [M − H]^−^ ion at *m*/*z* 473.1820. Its major diagnostic daughter ions were at *m*/*z* 237.1126, and 221.0819 a.m.u. Peak 47 and peak 52 were identified as α-collatolic acid and β-collatolic acid [29], which showed [M − H]^−^ ions at *m*/*z* 525.2130 for both. Their major diagnostic daughter ions were at *m*/*z* 263.1281 and 265.1076 a.m.u respectively.

***Dibenzofurans***: Strepsilin, with an [M − H]^−^ ion at *m/z* 269.0455, was evidenced as peak 12 [30]. The main daughter ions of peak 12 were at *m*/*z* 149.0238 and 123.0450 a.m.u. Peak 50 was identified as usnic acid [25] (molecular ion at *m/z* 343.0824). The main daughter ions of this peak were [M − H − CH_3_]^−^, [M − H − C_4_H_3_O_2_]^−^ and [M − H − C_5_H_3_O_3_]^−^ (328.0591, 259.0609 and 231.0661 a.m.u., respectively).

***Aromatic compounds***: Five simple aromatic compounds corresponding to the peaks 1, 10, 14, 19 and 37 were identified using UHPLC-DAD and HRMS–MS analysis. Peak 1 was identified as orsellinic acid [25], which showed an [M − H]^−^ ion at *m*/*z* 167.0345. The major diagnostic daughter MS ion of this compound was at *m*/*z* 123.0445 a.m.u. Peak 10 was identified as atranol (molecular anion at *m*/*z* 151.0395) whose fragmentation produced a diagnostic MS ion at *m*/*z* 123.0444. Peak 14 was assigned to haematommic acid [31] whose molecular anion was at *m*/*z* 195.0296. Its fragmentation produced ions at *m*/*z* 151.0390, 149.0240, and 123.0440 a.m.u. Peak 19 was identified as pentyldivaric acid, which showed an [M − H]^−^ ion at *m*/*z* 223.0974. Major diagnostic daughters MS ions of this peak were at *m*/*z* 167.0344, 149.0238 and 123.0445 a.m.u. Finally, ethylhaematommate was assigned to peak 37 (molecular anion at *m*/*z* 223.0610) whose fragmentation produced diagnostic MS ions at *m*/*z* 177.0189, 149.0238 and 123.0444 a.m.u.

***Chromones***: Lepraric acid (peak 31) was detected in the methanolic extract using this hyphenated technique [32]. Peak 31 showed an [M − H]^−^ ion at *m*/*z* 361.0931. Its major diagnostic daughter ions were at *m*/*z* 235.0606, 195.0292 and 149.0236 a.m.u.

### 2.2. Parmotrema Robustum

**Figure 2:**
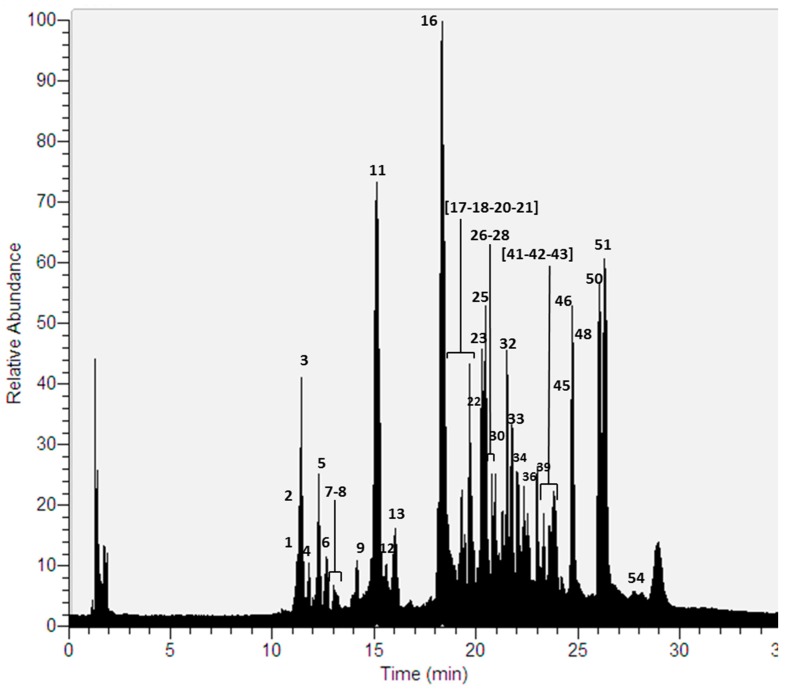
UHPLC Chromatogram of *P. robustum*.

Thirty-seven compounds (Figure 2) were detected for the first time in a methanolic extract of this species using UHPLC-ESI-MS-MS in negative mode (Table 1, Supplementary Materials Table S1). As in the previous case, the compounds were mainly depsides, depsidones, lipids, aromatics, diphenylethers and dibenzofurans [9,10,22,23,24,25,26].

***Depsides***: Thirteen depsides (peaks 6, 7, 9, 18, 30, 33, 39, 42, 43, 45, 46, 51 and 54) were identified. Peak 6 and 7 were identified as thamnolic acid and haemathamnolic acid, which showed [M − H]^−^ ions at *m*/*z* 419.0623 and 403.0675 respectively. Thamnolic acid produced major diagnostic MS ions at *m*/*z* 375.0722 and 167.0345 while haemathamnolic acid produced ions at *m*/*z* 209.0002 and 193.0503 u.m.a. Peak 30, with an [M − H]^−^ pseudomolecular ion at *m*/*z* 467.0984, was identified as gyrophoric acid, which showed diagnostic daughter ions at *m*/*z* 317.0667, 167.0344, 149.0240 and 123.0444. Peak 42 was identified as 4-O-methylgyrophoric acid based on its pseudomolecular ion at *m*/*z* 481.1141 and its daughter ions at *m*/*z* 317.0668, 167.0343, 149.0240 and 123.0443. Pseudocyphellarin A was assigned to peak 43 (molecular anion at *m*/*z* 401.1244). Major diagnostic daughter MS ions were at *m*/*z* 191.0347, 177.0552 and 133.0651 a.m.u. Peak 46 was identified as sekikaic acid (molecular anion at *m*/*z* 417.1559). The fragmentation of peak 46 produced ions at *m*/*z* 225.0768 [M − H − C_11_H_12_O_3_]^−^, 209.0814 [M − H − C_11_H_12_O_4_]^−^, and 165.0915 [M − H − C_12_H_12_O_5_]^−^. Peak 54 was identified as chloroatranorin [33], which showed an [M − H]^−^ ion at *m*/*z* 407.0542. The major diagnostic daughter ions were at *m*/*z* 228.9906, 210.9800 and 163.0394 a.m.u. Finally, peaks 18, 33, 45, and 51 were identified as lecanoric acid, evernic acid, barbatic acid and atranorin respectively.

***Depsidones***: Thirteen depsidones corresponding to the peaks 2, 4, 5, 8, 11, 13, 16, 17, 22, 23, 25, 41 and 48 were identified in this species. Peak 2 was identified as consalazinic acid [34] which showed an [M − H]^−^ ion at *m*/*z* 389.0517. Its major diagnostic daughter ions were at *m*/*z* 371.0479, 309.0406, 253.0506 and 209.0605 a.m.u. Peaks 4, 8 and 13 were identified as conzalasinic acid derivatives based on both their pseudomolecular ions and daughter ions. Conprotocetraric acid [35] was at peak 5 (molecular anion at *m*/*z* 375.0724) and their fragmentation produced ions at *m*/*z* 357.0610, 313.0722, 295.0618 and 251.0710. Peak 17, with a [M − H]^−^ ion at *m*/*z* 401.0516, was identified as a constictic acid derivative. The parent ion produced major diagnostic MS ions at *m*/*z* 373.0568, 357.0618, and 151.0392, confirming this derivative, unlike constictic acid whose daughter ions were at *m*/*z* 373.0565, 357.0614, 283.0601, and 227.0698. Peak 23, with a [M − H]^−^ pseudomolecular ion at *m*/*z* 371.0412, was identified as norstictic acid [36], which showed diagnostic daughter ions at *m*/*z* 283.0616, 267.0667, 243.0292 and 227.0348. α-alectoronic acid was assigned to peak 41 (molecular anion at *m*/*z* 511.1973). The fragmentation of peak 41 produced ions at *m*/*z* 467.2050, 369.1338 and 247.0974. Finally, peaks 11, 16, 22, 25 and 48 were identified as salazinic acid, connorstictic acid, substictic acid, hypoconstictic and lobaric acid [36], respectively.

***Lipids***: Five polyhydroxylated lipids were tentatively identified (peaks 20, 21, 26, 28, and 32) using UHPLC–ESI–MS–MS analyses [28]. Peak 20, with an [M − H]^−^ ion at *m*/*z* 447.3329, was tentatively identified as pentahydroxytetracosanoic acid. Peaks 26, 28 and 32 showed [M − H]^−^ ions at *m*/*z* 431.3380, 475.3635 and 493.3383, and were tentatively identified as tetrahydroxytetracosanoic acid, pentahydroxyhexacosanoic acid and heptahydroxypentacosanoic acid, respectively. As indicated above, peak 21 was identified as tetrahydroxydocosanoic acid.

***Diphenylethers***: A diphenylether was detected in this species. Peak 36 was identified as β-alectoronic acid [7], which showed an [M − H]^−^ ion at *m*/*z* 511.1974. Its major diagnostic daughter ions were at *m*/*z* 369.1339, 247.0967 and 163.0396 a.m.u.

***Dibenzofurans***: Strepsilin (peak 12) and usnic acid (peak 50) were identified in this species, as indicated above.

***Aromatic compounds***: An aromatic compound corresponding to peak 1 was identified in this analysis. As indicated above, peak 1 was identified as orsellinic acid [26].

***Unknown compounds***: Two compounds (peaks 3 and 34) were not identified.

**Figure 3:**
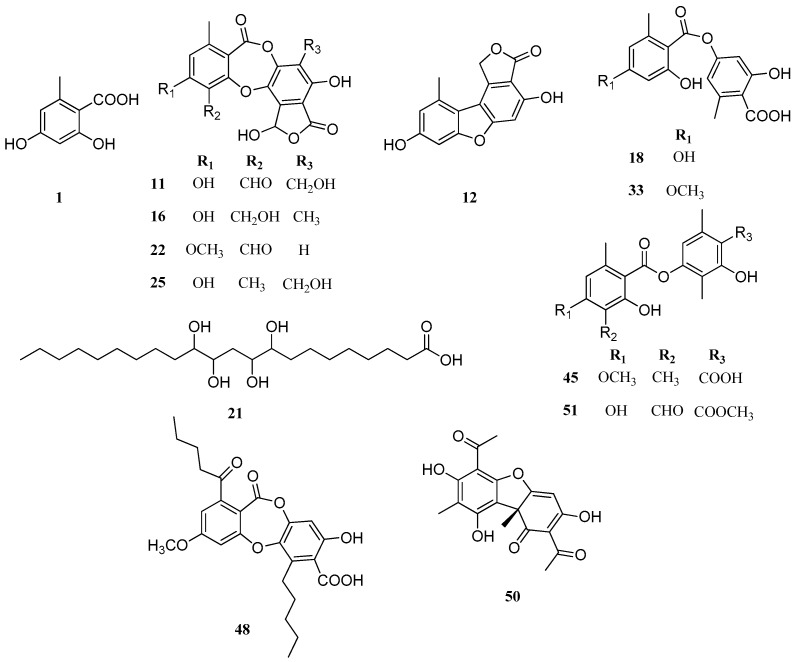
Chemical structures of similar compounds in *P. andinum* and *P. robustum*.

Thirteen compounds (Figure 3) were detected in both *Parmotrema* species, which correspond to orsellinic acid (peak 1), salazinic acid (peak 11), strepsilin (peak 12), connorstictic acid (peak 16), lecanoric acid (peak 18), tetrahydroxydocosanoic acid (peak 21), substictic acid (peak 22), hypoconstictic acid (peak 25), evernic acid (peak 33), barbatic acid (peak 45), lobaric acid (peak 48), usnic acid (peak 50) and atranorin (peak 51). *P. robustum* produced more depsides and depsidones than *P. andinum*, while the latter is more a producer of aromatic compounds. Also, in *P. andinum* a chromone (peak 31) was assigned to lepraric acid. According to SciFinder, there are no chemical studies on *P. andinum* and *P. robustum*. Therefore, our work represents the first study on the chemistry of these *Parmotrema* species.

## 3. Materials and Methods

### 3.1. Lichen Material

The lichen specimen *Parmotrema andinum* (*Müll. Arg*.) Hale (30 g) was collected at “Huaraz,” Ancash, Peru, in 2015. A voucher specimen, PA-61-USM-2015, was deposited in the Museo de Historia Natural de la UNMSM, and Prof. Dr. Haydee Montoya confirmed its identity.

The species *Parmotrema robustum* (Degel.) (17 g) was collected in “Combeima river basin,” Ibagué-Tolima, Colombia by M. Rivera-Montalvo and Prof. A. Torres-Benítez. A voucher specimen, COL-011, was deposited in the herbarium of Universidad Distrital Francisco José de Caldas, and Prof. Alejandra Suárez Corredor confirmed its identity.

### 3.2. UHPLC-Orbitrap-ESI-MS-MS

Sample preparation: Some 3 grams of each lichen were macerated with methanol (3 times, 30 mL each time, 3 days/extraction). The solutions were concentrated to obtain 19 mg (*P. andinum*), and 11 mg (*P. robustum*) of a gummy extract.

#### 3.2.1. Instrument

A Thermo Scientific Dionex Ultimate 3000 UHPLC system, equipped with a quaternary Series RS pump and Thermo Scientific Dionex Ultimate 3000 Series TCC-3000RS column compartments with a Thermo Fisher Scientific Ultimate 3000 Series WPS-3000RS autosampler and a rapid separations PDA detector controlled by Chromeleon 7.2 Software (Thermo Fisher Scientific, Waltham, MA, USA and Dionex Softron GmbH Part of Thermo Fisher Scientific, Germering, Germany) hyphenated with a Thermo high resolution Q Exactive focus mass spectrometer (Thermo, Bremen, Germany) were used for the analysis. The chromatographic system was coupled to the MS with a Heated Electrospray Ionization Source II (HESI II). Nitrogen (purity > 99.999%) obtained from a Genius NM32LA nitrogen generator (Peak Scientific, Billerica, MA, USA) was employed as both the collision and damping gas. Mass calibration for Orbitrap was performed once a week, in both negative and positive modes, to ensure a working mass accuracy lower than or equal to 5 ppm. Caffeine and N-butylamine (Sigma Aldrich, Saint Louis, MO, USA) were the calibration standards for positive ions and buspirone hydrochloride, sodium dodecyl sulfate, and taurocholic acid sodium salt (Sigma Aldrich, Saint Louis, MO, USA) were used to calibrate the mass spectrometer. These compounds were dissolved in a mixture of acetic acid, acetonitrile, water and methanol (Merck, Darmstadt, Germany) and were infused using a Chemyx Fusion 100 syringe pump (Thermo Fisher Scientific, Bremen, Germany). XCalibur 2.3 software (Thermo Fisher Scientific, Bremen, Germany) and Trace Finder 3.2 (Thermo Fisher Scientific, San José, CA, USA) were used for UHPLC control and data processing, respectively. Q Exactive 2.0 SP 2 from Thermo Fisher Scientific was used to control the mass spectrometer.

#### 3.2.2. LC Parameters

An UHPLC C18 column (Acclaim, 150 mm × 4.6 mm ID, 5 m, Thermo Fisher Scientific, Bremen, Germany) operated at 25 °C was employed. The detection wavelengths were 254, 280, 320 and 440 nm. PDA was recorded from 200 to 800 nm, and mobile phases were 1% formic aqueous solution (A) and acetonitrile (B). The gradient program (time (min), % B) was: (0.00, 5); (5.00, 5); (10.00, 30); (15.00, 30); (20.00, 70); (25.00, 70); (35.00, 5) and 12 min for column equilibration before each injection. The flow rate was 1.00 mL min^−1^, and the injection volume was 10 µL. Standards and lichen extracts dissolved in methanol were kept at 10 °C inside the autosampler.

#### 3.2.3. MS Parameters

The HESI parameters were as follows: sheath gas flow rate, 75 units; auxiliary gas unit flow rate, 20; capillary temperature, 400 °C; auxiliary gas heater temperature, 500 °C; spray voltage, 2500 V (for ESI-); and S lens, RF level 30. Full scan data in positive and negative were acquired at a resolving power of 70,000 FWHM (full width half maximum) at m/z 200. For the compounds of interest, a scan range of *m*/*z* 100–1000 was chosen; the automatic gain control (AGC) was set at 3 × 10^6^ and the injection time was set to 200 ms. The scan-rate was set at 2 scans s^−1^. External calibration was performed using a calibration solution in positive and negative modes. For confirmation purposes, a targeted MS-MS analysis was performed using the mass inclusion list, with a 30 s time window, with the Orbitrap spectrometer operating both in positive and negative modes at 17,500 FWHM (*m*/*z* 200). The AGC target was set to 2 × 10^5^, with the maximum injection time of 20 ms. The precursor ions were filtered by the quadrupole, which operated at an isolation window of *m*/*z* 2. The fore vacuum, high vacuum and ultrahigh vacuum were maintained at approximately 2 mbar, from 105 and below 1010 mbar, respectively. Collision energy (HCD cell) was operated at 30 kv. Detection was based on calculated exact mass and on retention time of target compounds, as shown in Table 1. The mass tolerance window was set to 5 ppm for the two modes.

## 4. Conclusions

In the present report, fifty-four metabolites were detected using UHPLC-Q-Orbitrap-ESI-MS-MS for the first time in *P. andinum* and *P. robustum*. Our study indicates that lipids, depsides, depsidones, dibenzofurans, diphenylethers and aromatic compounds were the main compounds detected and identified. This report could contribute to the better understanding of the chemistry of *Parmotrema* genus, and it supports that the HPLC fingerprints are very important for the fast chemical differentiation of these ruffle lichens.

## Figures and Tables

**Table 1:** Identification of lichen substances in *Parmotrema* species by UHPLC-ESI-MS-MS.

Peak	Tentative Identification	[M − H]^−^	Retention Time (min)	Theoretical Mass (*m/z*)	Measured Mass (*m/z*)	Accuracy (ppm)	Metabolite Type	MS^2^ Ions (ppm)	Lichens
1	Orsellinic acid	C_8_H_7_O_4_	11.32	167.0344	167.0345	0.6	A	123.0445	PA; PR
2	Consalazinic acid	C_18_H_13_O_10_	11.41	389.0514	389.0517	0.8	D	371.0409; 309.0406; 253.0506; 209.0605	PR
3	Unknown	C_22_H_18_O_11_N	11.81	472.0880	472.0888	1.7	-	-	PR
4	Consalazinic acid derivative	C_18_H_11_O_11_	12.02	403.0307	403.0312	1.2	D	387.0363; 385.0207; 329.0303; 149.0244; 253.0497; 241.0511	PR
5	Conprotocetraric acid	C_18_H_15_O_9_	12.27	375.0716	375.0724	2.1	D	357.0610; 313.0722; 295.0618; 251.0710	PR
6	Thamnolic acid	C_19_H_15_O_11_	12.66	419.0614	419.0623	2.1	d	375.0722; 167.0345	PR
7	Haemathamnolic acid	C_19_H_15_O_10_	13.03	403.0665	403.0675	2.5	hd	209.0002; 193.0503	PR
8	Consalazinic acid derivative	C_19_H_13_O_11_	13.23	417.0463	417.0467	0.9	D	387.0358; 327.0513; 239.0351; 177.0193	PR
9	Squamatic acid	C_19_H_17_O_9_	14.16	389.0873	389.0875	0.5	d	211.0260	PR
10	Atranol	C_8_H_7_O_3_	14.72	151.0395	151.0395	0.0	A	123.0444	PA
11	Salazinic acid	C_18_H_11_O_10_	15.03	387.0357	387.0359	0.5	D	243.03078; 227.0343; 151.0394; 121.0291	PA; PR
12	Strepsilin	C_15_H_9_O_5_	15.58	269.0450	269.0455	1.9	DPB	149.0238; 123.0450	PA; PR
13	Consalazinic acid derivative	C_20_H_17_O_11_	16.04	433.0776	433.0778	0.5	D	401.0516; 387.0360; 343.0462; 269.0457	PR
14	Haematommic acid	C_9_H_7_O_5_	16.49	195.0293	195.0296	1.5	A	151.0390; 149.0240; 123.0440	PA
15	Stictic acid	C_19_H_13_O_9_	18.37	385.0560	385.0568	2.1	D	341.0668; 297.0760; 267.0297; 165.0544	PA
16	Connorstictic acid	C_18_H_13_O_9_	18.53	373.0560	373.0568	2.0	D	329.0665; 181.0554	PA; PR
17	Constictic acid derivative	C_19_H_13_O_10_	19.22	401.0509	401.0516	1.7	D	373.0568; 357.0618; 151.0392	PR
18	Lecanoric acid	C_16_H_13_O_7_	19.40	317.0661	317.0668	2.2	d	167.0342; 149.0236; 123.0445	PA; PR
19	pentyldivaric acid	C_12_H_15_O_4_	19.66	223.0970	223.0974	1.8	A	167.0344; 149.0238; 123.0445	PA
20	Pentahydroxytetracosanoic acid	C_24_H_47_O_7_	19.68	447.3327	447.3329	0.4	L	-	PR
21	Tetrahydroxydocosanoic acid	C_22_H_43_O_6_	19.84	403.3065	403.3069	0.8	L	-	PA; PR
22	Substictic acid	C_18_H_11_O_9_	20.00	371.0403	371.0411	2.2	D	327.0512; 255.0660	PA; PR
23	Norstictic acid	C_18_H_11_O_9_	20.08	371.0403	371.0412	2.1	D	283.0616; 267.0667; 243.0292; 227.0348	PR
24	Decarboxythamnolic acid	C_18_H_15_O_9_	20.16	375.0721	375.0724	0.8	d	209.0450; 167.0345; 139.0394	PA
25	Hypoconstictic acid	C_19_H_15_O_9_	20.60	387.0716	387.0724	2.1	D	343.0825; 299.0921	PA; PR
26	Tetrahydroxytetracosanoic acid	C_24_H_47_O_6_	20.74	431.3378	431.3380	0.5	L	-	PR
27	Loxodinol	C_25_H_29_O_9_	20.90	473.1812	473.1820	2.1	DPE	237.1126; 221.0819	PA
28	Pentahydroxyhexacosanoic acid	C_26_H_51_O_7_	20.95	475.3640	475.3635	1.1	L	-	PR
29	Pentahydroxyoxooctacosanoic acid	C_28_H_53_O_8_	21.29	517.3746	517.3748	0.3	L	-	PA
30	Gyrophoric acid	C_24_H_19_O_10_	21.30	467.0978	467.0984	1.3	d	317.0666; 167.0344; 149.0240; 123.0444	PR
31	Lepraric acid	C_18_H_17_O_8_	21.66	361.0923	361.0931	2.2	C	235.0606; 195.0292; 149.0236	PA
32	Heptahydroxypentacosanoic acid	C_25_H_49_O_9_	21.72	493.3382	493.3383	0.2	L	-	PR
33	Evernic acid	C_17_H_15_O_7_	21.76	331.0818	331.0825	2.1	d	167.0345; 149.0238; 123.0444	PA; PR
34	Unknown	C_29_H_27_O_13_	22.04	583.1457	583.1453	0.7	-	253.0504; 163.0394; 119.0495	PR
35	Furfuric acid	C_28_H_23_O_12_	22.44	551.1190	551.1194	0.7	D	359.1130; 179.0345; 163.0394; 137.0601	PA
36	β-Alectoronic acid	C_28_H_31_O_9_	22.55	511.1968	511.1974	1.2	DPE	369.1339; 247.0967; 163.0396	PR
37	Ethyl haematommate	C_11_H_11_O_5_	22.66	223.0606	223.0610	1.8	A	177.0189; 149.0238; 123.0444	PA
38	Hydroxydioxohenicosanoic acid	C_21_H_37_O_5_	22.96	369.2641	369.2649	2.2	L	-	PA
39	Methyl-3’-methyl lecanorate	C_18_H_17_O_7_	23.00	345.0980	345.0983	0.8	d	167.0344; 149.0238; 123.0444	PR
40	Trioxohenicosanoic acid	C_21_H_35_O_5_	23.25	367.2490	367.2492	2.2	L	-	PA
41	α-Alectoronic acid	C_28_H_31_O_9_	23.45	511.1968	511.1973	1.2	D	467.2050; 369.1338; 247.0974	PR
42	4-*O*-Methylgyrophoric acid	C_25_H_21_O_10_	23.63	481.1135	481.1141	1.2	d	317.0668; 167.0343; 149.0240; 123.0443	PR
43	Pseudocyphellarin A	C_21_H_21_O_8_	23.86	401.1236	401.1244	2.0	d	191.0347; 177.0552; 133.0651	PR
44	2-*O*-Methylstenosporic acid	C_24_H_29_O_7_	23.88	429.1913	429.1922	2.1	d	223.0972; 179.1072	PA
45	Barbatic acid	C_19_H_19_O_7_	24.24	359.1131	359.1138	1.9	d	181.0501; 163.0394; 137.0600	PA; PR
46	Sekikaic acid	C_22_H_25_O_8_	24.34	417.1542	417.1559	4.1	d	225.0768; 209.0814; 165.0915; 150.0680	PR
47	α-Collatolic acid	C_29_H_33_O_9_	24.66	525.2125	525.2130	1.0	DPE	263.1281	PA
48	Lobaric acid *	C_25_H_27_O_8_	24.96	455.1711	455.1712	0.2	D	411.1815; 367.1909; 352.1681	PA; PR
49	Dihydroxydioxononadecanoic acid	C_19_H_33_O_6_	25.40	357.2277	357.2285	2.2	L	-	PA
50	Usnic acid *	C_18_H_15_O_7_	26.13	343.0818	343.0824	1.7	DBF	328.0591; 259.0609; 231.0661	PA; PR
51	Atranorin	C_19_H_17_O_8_	26.33	373.0923	373.0930	1.9	d	177.0190; 163.0386	PA; PR
52	β-Collatolic acid	C_29_H_33_O_9_	26.81	525.2125	525.2130	1.0	DFE	265.1076	PA
53	Dihydroxyheptadecatrienoic acid	C_17_H_27_O_4_	27.41	295.1909	295.1917	2.7	L	-	PA
54	Chloroatranorin	C_19_H_16_ClO_8_	28.93	407.0534	407.0542	2.0	d	228.9906; 210.9800; 163.0394	PR

* Identified by spiking experiments with an authentic compound. A = Aromatic; L = Lipid; D = depsidone; d = depside; DPE = diphenylether; DBF = dibenzofuran. C = Chromone; PA: *Parmotrema andinum*; PR: *Parmotrema robustum*; MS^2^ = Daughter ions.

## Supplementary Materials

The following are available online, Table S1: Structure of the compounds identified by UHPLC-ESI-MS-MS from *Parmotrema* species.
