# Supplementary material for: Metabolomic Analysis of Two Parmotrema Lichens: P. robustum (Degel.) Hale and P. andinum (Mull. Arg.) Hale Using UHPLC-ESI-OT-MS-MS
Source: Molecules. 2017 Oct 30;22(11):1861. doi: 10.3390/molecules22111861 (PMC6150355; doi:10.3390/molecules22111861)
Supplement: Supplementary file 1 [file molecules-22-01861-s001.pdf]

molecules

MDPI

Supporting Data

Metabolomic Analysis of Two Lichens: *Parmotrema robustum* (Degel.) Hale and *Parmotrema andinum* (Mull. Arg.) Hale Using UHPLC-ESI-OT-MS-MS

Alfredo Torres-Benítez, María Rivera-Montalvo, Beatriz Sepúlveda, Olivio N. Castro, Edgar Nagles, Mario J. Simirgiotis, Olimpo García-Beltrán and Carlos Areche

Table S1. Structure of the compounds identified by UHPLC-ESI-MS-MS from *Parmotrema* species.

Number	Name	Structure
1	Orsellinic acid	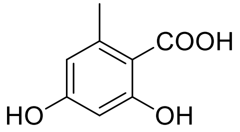
2	Consalazinic acid	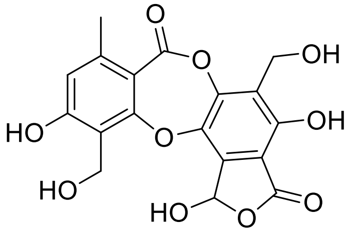
5	Conprotocetraric acid	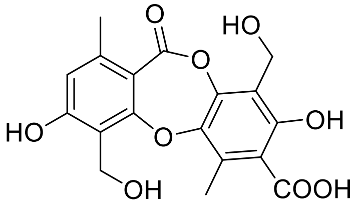

6	Thamnolic acid	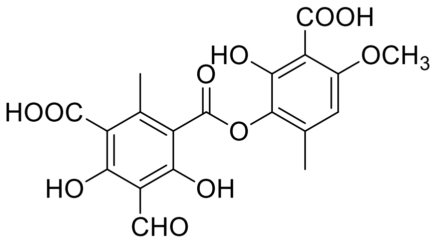
7	Haemathamnolic acid	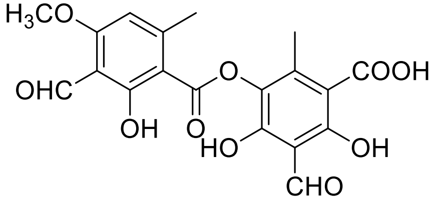
9	Squamatic acid	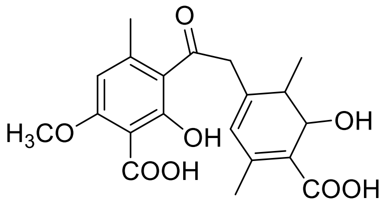
10	Atranol	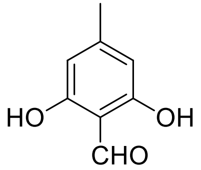
11	Salazinic acid	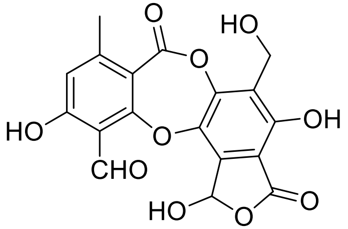

12	Strepsilin	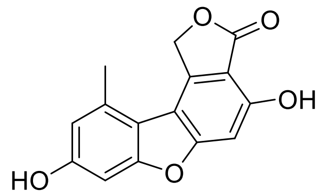
14	Haematommic acid	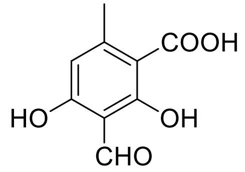
15	Stictic acid	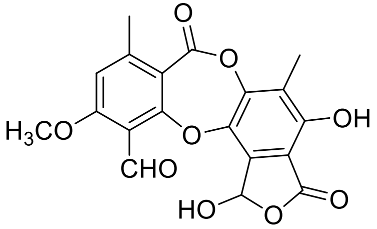
16	Connorstictic acid	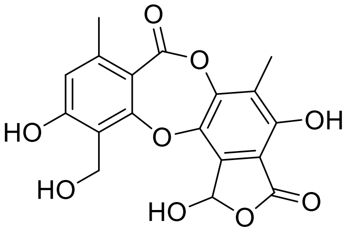
18	Lecanoric acid	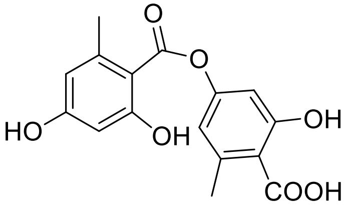

19	Pentyldivaric acid	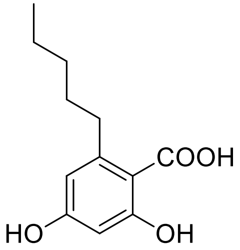
20	Pentahydroxytetracosanoic acid	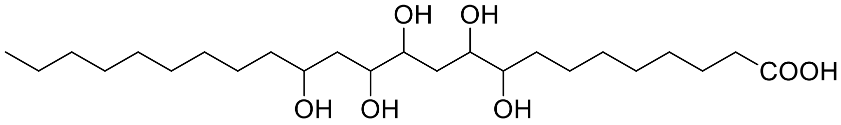
21	9,10,12,13-tetrahydroxydocosanoic acid	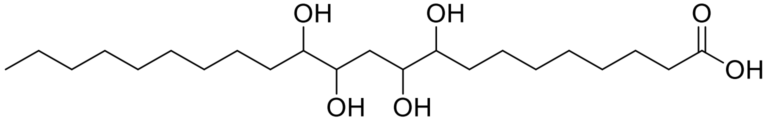
22	Substictic acid	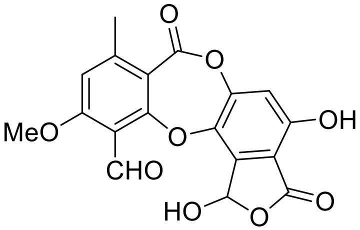
23	Norstictic acid	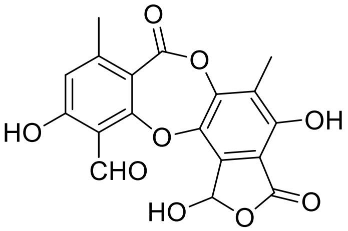
24	Decarboxythamnolic acid	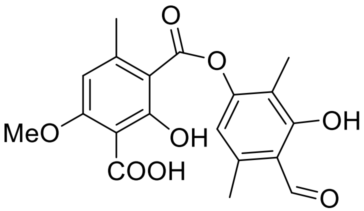

25	Hypoconstictic acid	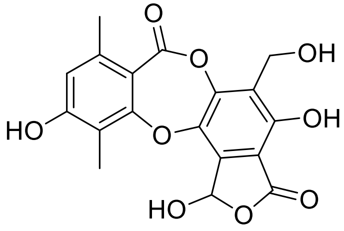
26	Tetrahydroxytetracosanoic acid	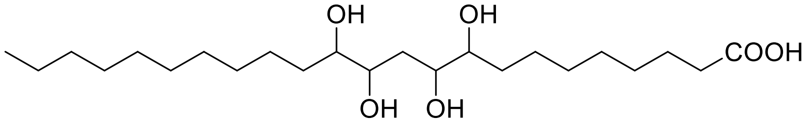
27	Loxodinol	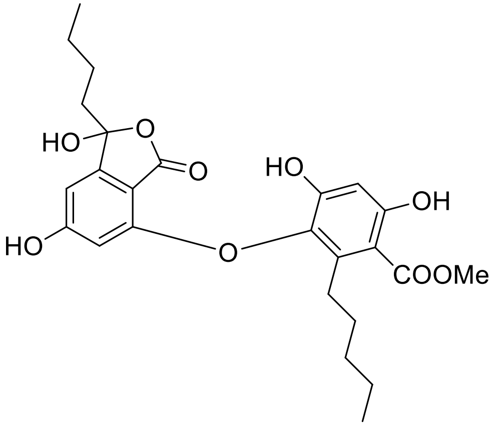
28	Pentahydroxyhexacosanoic acid	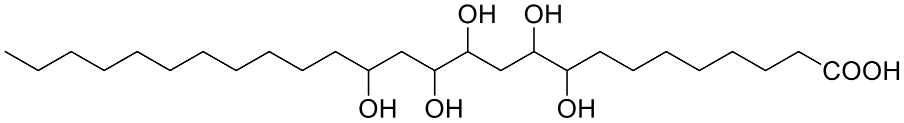
29	Pentahydroxyoxooctacosanoic acid	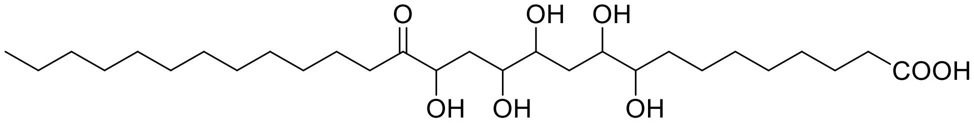

30	Gyrophoric acid	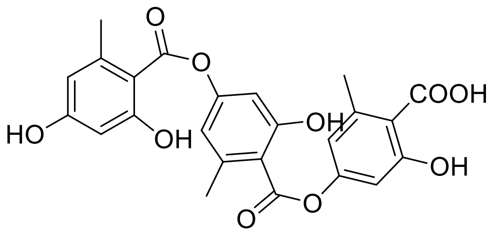
31	Lepraric acid	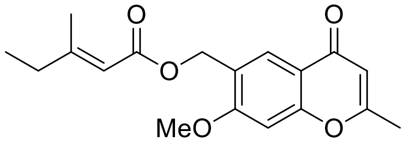
32	Heptahydroxypentacosanoic acid	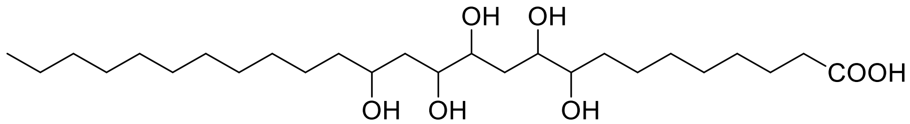
33	Evernic acid	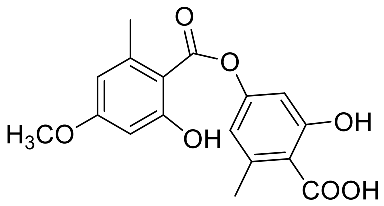
35	Furfuric acid	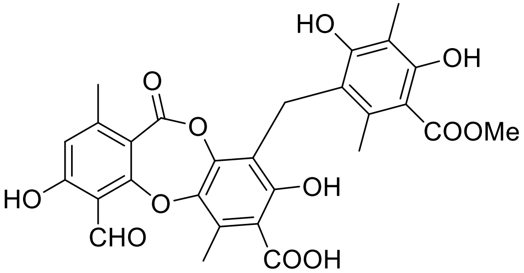

36	β-Alectoronic acid	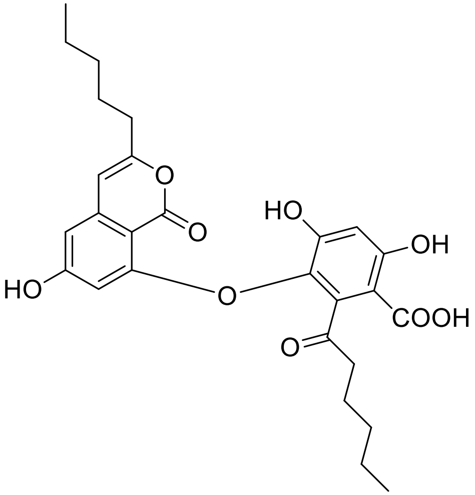
37	Ethyl haematommate	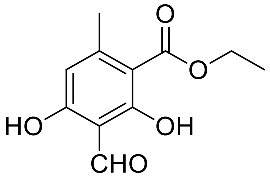
38	Hydroxydioxohenicosanoic acid	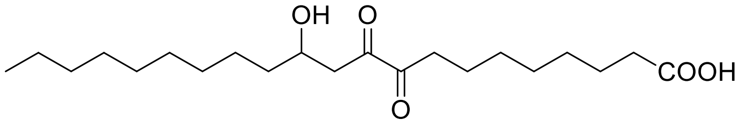
39	Methyl-3′-methyl lecanorate	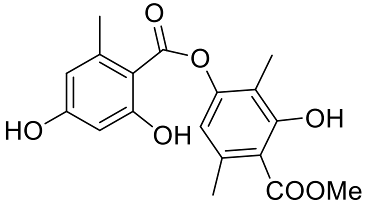
40	Trioxohenicosanoic acid	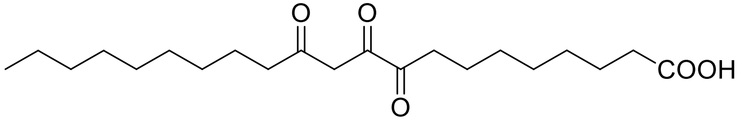

41	α-Alectoronic acid	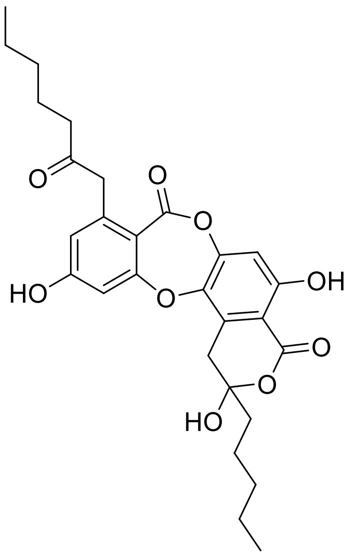
42	4-O-Methylgyrophoric acid	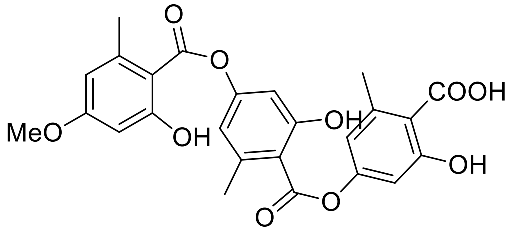
43	Pseudocyphellarin A	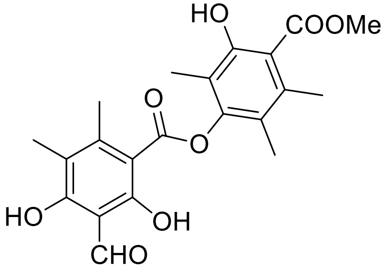

44	2-0-Methylstenosporic acid	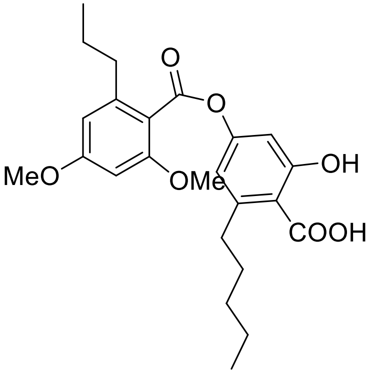
45	Barbatic acid	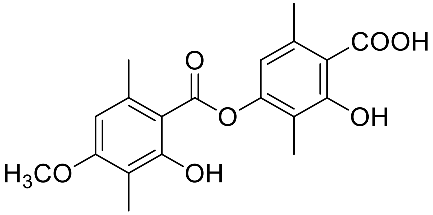
46	Sekikaic acid	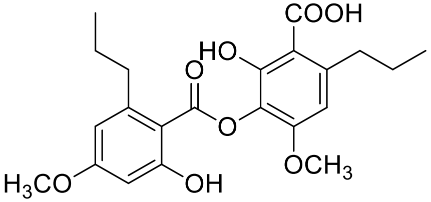

47	α-Collatolic acid	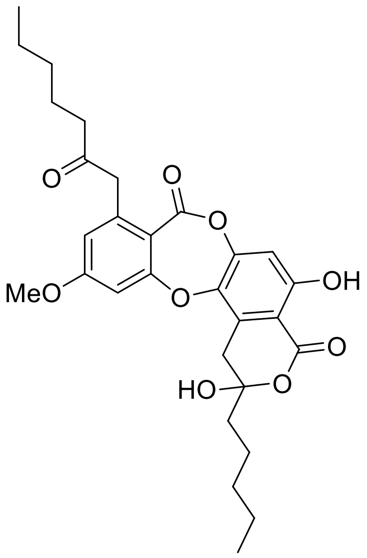
48	Lobaric acid	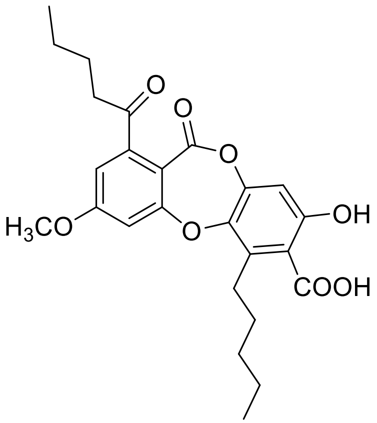
49	Dihydroxydioxononadecanoic acid	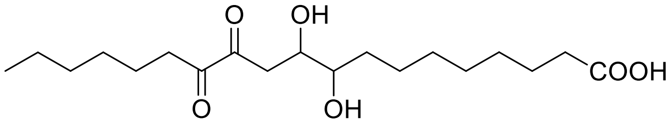

50	Usnic acid	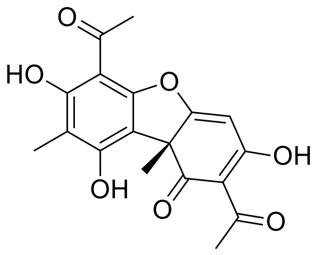
51	Atranorin	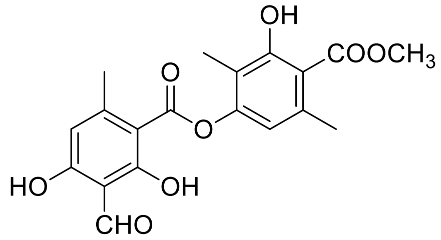
52	β-Collatolic acid	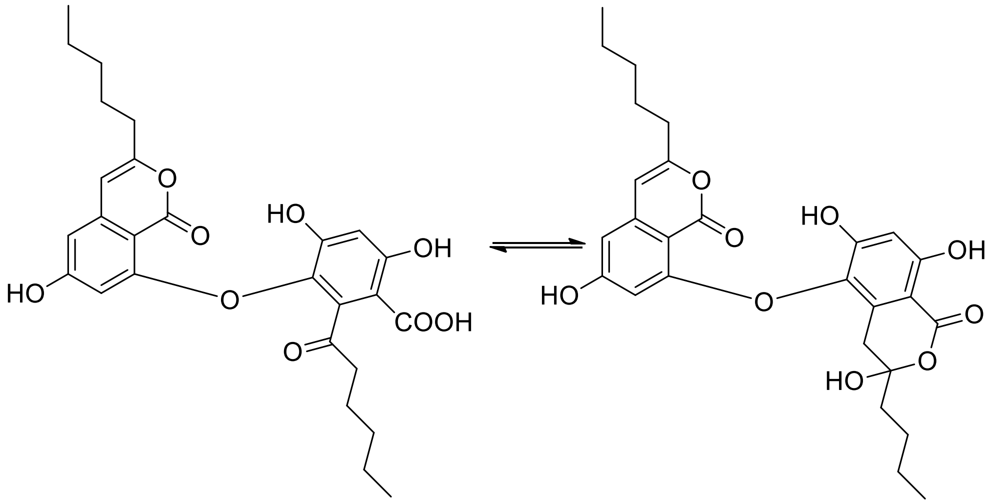
53	Dihydroxyheptadecatrienoic acid	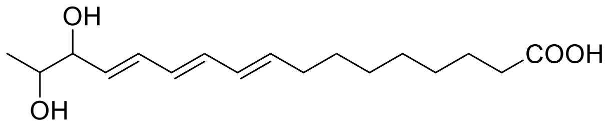

54	Chloroatranorin	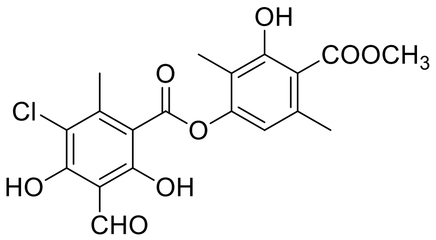
